# Wound Repair and Regeneration: Mechanisms, Signaling

**DOI:** 10.3390/ijms20246328

**Published:** 2019-12-15

**Authors:** Sadanori Akita

**Affiliations:** Department of Plastic Surgery, Wound Repair and Regeneration, Fukuoka University, School of Medicine, 7-45-1 Nanakuma, Jonan-ku, Fukuoka 8140180, Japan; akitas@hf.rim.or.jp

Wound healing plays an integral part of cellular and molecular events. This process may be implicated in tissue regeneration. Regeneration can be contributed to complete tissue restoration and improvement of tissue disfigurement towards the original condition. Also, such cellular and molecular events are orchestrated both spatially and temporally. 

Tissue regeneration, scar-less wound healing, and fibrosis are all dependent upon the phylogenetic event of the organism, as well as the inflammatory responses, which are influenced by age, sex, and interaction with the environment [1]. Under these conditions, the lack of a true blastema allows for only scarring wound repair in the inbred MRL/MpJ strain of mice and the outbred CD-1 and Swiss Webster laboratory mouse stocks [2].

In cytokines, IL-1 and TNF-α are always present during wound repair, but their pleiotropic and synergistic effects are not well understood. Rather than improving wound repair in young males, IL-1 signaling blockade increased epithelial thickness and IL-1β and TNF-α expression, and diminished epidermal apoptosis. TNF-α impaired wound repair in middle-aged females, which exhibited acanthosis and overexpression of IL-1, but no change in apoptosis. These findings suggest that this mechanism of epidermal thickening differs from that observed in IL1-ra-treated animals [3].

In this issue, Aoki et al. report a sphingosine-1-phosphate (S1P), which is a lipid mediator that promotes angiogenesis, cell proliferation, and attracts immune cells. They clarify the roles of S1P in skin wound healing by altering the expression of its biogenic enzyme, sphingosine kinase-1 (SphK1). The SphK1 overexpression also leads to less scarring, and the interaction between transforming growth factor (TGF)-β1 and S1P receptor-2 (S1PR2) signaling is likely to play a key role [4].

Kanno et al. find an interferon (IFN)-γ, known for its inhibitory effects on collagen synthesis by fibroblasts in vitro; however, information is limited regarding its role in wound healing in vivo. IFN-γ might be involved in the proliferation and maturation stages of wound healing through the regulation of neutrophilic inflammatory responses in IFN-γ-deficient (KO) mice [5]. 

Wound impairment is accelerated and completed with the local administration of recombinant human (rh)-growth hormone (GH) accelerating PU healing in non-obese diabetic/severe combined immunodeficient mice engrafted with a full-thickness human skin graft model in 60 days [6].

Other than skin, matrisome properties of scaffolds directing fibroblasts in idiopathic pulmonary fibrosis [7] and liver regeneration are enhanced by hepatocyte-derived angiogenesis via B-cell CLL/lymphoma/nuclear factor-Kappa B signaling [8], while wound repair and regeneration mechanisms of autologous adipose-derived stem cells in some patients with human immunodeficiency virus (HIV), treated by highly active antiretroviral therapy, are elucidated and analyzed in detail [9].

In novel aspects, the cloning and identification of Periplaneta americana, the American cockroach, thymosin (Pa-THYs) are obtained by bioinformatics and it is found that Pa-THYs also stimulate the expression of several key growth factors to promote wound healing. The data suggest that Pa-THYs could be a potential drug for promoting wound repair [10]. 

Lastly, maresins (MaRs) and macrophages are reviewed, focusing on the potent action of MaRs to enhance M2 macrophage phenotypic profiles to possibly alleviate inflammatory pain [11].

## References

[1] Eming S.A., Martin P., Tomic-Canic M. (2014). Wound repair and regeneration: Mechanisms, signaling, and translation. Sci. Transl. Med..

[2] Gawriluk T.R., Simkin J., Thompson K.L., Biswas S.K., Clare-Salzler Z., Kimani J.M., Kiama S.G., Smith J.J., Ezenwa V.O., Seifert A.W. (2016). Comparative analysis of ear-hole closure identifies epimorphic regeneration as a discrete trait in mammals. Nat. Commun..

[3] Abarca-Buis R.F., Martínez-Jiménez A., Vera-Gómez E., Contreras-Figueroa M.E., Garciadiego-Cázares D., Paus R., Robles-Tenorio A., Krötzsch E. (2018). Mechanisms of epithelial thickening due to IL-1 signalling blockade and TNFα administration differ during wound repair and regeneration. Differentiation.

[4] Aoki M., Aoki H., Mukhopadhyay P., Tsuge T., Yamamoto H., Matsumoto N.M., Toyohara E., Okubo Y., Ogawa R., Takabe K. (2019). Sphingosine-1-Phosphate Facilitates Skin Wound Healing by Increasing Angiogenesis and Inflammatory Cell Recruitment with Less Scar Formation. Int. J. Mol. Sci..

[5] Kanno E., Tanno H., Masaki A., Sasaki A., Sato N., Goto M., Shisai M., Yamaguchi K., Takagi N., Shoji M. (2019). Defect of Interferon γ Leads to Impaired Wound Healing through Prolonged Neutrophilic Inflammatory Response and Enhanced MMP-2 Activation. Int. J. Mol. Sci..

[6] Cristóbal L., de los Reyes N., Ortega M.A., Álvarez-Mon M., García-Honduvilla N., Buján J., Maldonado A.A. (2019). Local Growth Hormone Therapy for Pressure Ulcer Healing on a Human Skin Mouse Model. Int. J. Mol. Sci..

[7] Rendin L.E., Löfdahl A., Åhrman E., Müller C., Notermans T., Michaliková B., Rosmark O., Zhou X.-H., Dellgren G., Silverborn M. (2019). Matrisome Properties of Scaffolds Direct Fibroblasts in Idiopathic Pulmonary Fibrosis. Int. J. Mol. Sci..

[8] Chou C.-H., Ho C.-M., Lai S.-L., Chen C.-N., Wu Y.-M., Shun C.-T., Wen W.-F., Lai H.-S. (2019). B-Cell Activating Factor Enhances Hepatocyte-Driven Angiogenesis via B-Cell CLL/Lymphoma 10/Nuclear Factor-KappaB Signaling during Liver Regeneration. Int. J. Mol. Sci..

[9] Suzuki K., Akita S., Yoshimoto H., Ohtsuru A., Hirano A., Yamashita S. (2019). Biological Features Implies Potential Use of Autologous Adipose-Derived Stem/Progenitor Cells in Wound Repair and Regenerations for the Patients with Lipodystrophy. Int. J. Mol. Sci..

[10] Jing J., Sun X., Zhou C., Zhang Y., Shen Y., Zeng X., Yue B., Zhang X. (2019). Cloning, Expression and Effects of *P. americana* Thymosin on Wound Healing. Int. J. Mol. Sci..

[11] Hwang S.-M., Chung G., Kim Y.H., Park C.-K. (2019). The Role of Maresins in Inflammatory Pain: Function of Macrophages in Wound Regeneration. Int. J. Mol. Sci..

